# Revisiting the Rate-Dependent Mechanical Response of Typical Silicon Structures via Molecular Dynamics

**DOI:** 10.3390/nano12071203

**Published:** 2022-04-03

**Authors:** Yi Liu, Wei Wan, Quan Li, Zhenkang Xiong, Changxin Tang, Lang Zhou

**Affiliations:** 1 Institute of Photovoltaics, Nanchang University, Nanchang 330031, China; slouis@ncu.edu.cn (Y.L.); lzhou@ncu.edu.cn (L.Z.); 2 Institute for Advanced Study, Nanchang University, Nanchang 330031, China; 3 School of Automotive Application, Hunan Automotive Engineering Vocational College, Zhuzhou 412001, China; leewelling@163.com; 4 School of Material Science and Engineering, Nanchang University, Nanchang 330031, China; 5 Department of Science and Technology, Nanchang University College of Science and Technology, Jiujiang 332020, China; k1242638322@163.com

**Keywords:** molecular dynamics, monocrystalline silicon, silicon nanowire, strain rate, mechanical response

## Abstract

Strain rate is a critical parameter in the mechanical application of nano-devices. A comparative atomistic study on both perfect monocrystalline silicon crystal and silicon nanowire was performed to investigate how the strain rate affects the mechanical response of these silicon structures. Using a rate response model, the strain rate sensitivity and the critical strain rate of two structures were given. The rate-dependent dislocation activities in the fracture process were also discussed, from which the dislocation nucleation and motion were found to play an important role in the low strain rate deformations. Finally, through the comparison of five equivalent stresses, the von Mises stress was verified as a robust yield criterion of the two silicon structures under the strain rate effects.

## 1. Introduction

Monocrystalline silicon (Mono-Si) has attracted great attention in recent decades due to its material properties and promising applications in the field of semiconductors, photovoltaics and Micro Electro Mechanical Systems [1,2]. Among these attractive properties, mechanical performance is crucial for future applications of silicon-based devices because it is a prerequisite to fulfill other functionalities. Although perfect Mono-Si crystals can be produced through Floating Zone [3] or Czochralski [4] methods, traditional mechanical experiments are still difficult to execute due to the inherent brittleness of silicon. Moreover, the produced Mono-Si wafers have also exhibited edge collapse, hidden cracks and subsurface damage [5,6] in wiresaw cutting, which have slowed its commercial expansion in the photovoltaic industry. Therefore, learning the mechanical properties of silicon is still highly desirable, more and more efforts have been continuously carried out to solve these problems.

Among the relevant studies about the mechanical properties of silicon, a particularly intriguing question is how the applied strain rate affects its mechanical performance, fracture behaviors and deformation mechanisms. The corresponding solutions will not only deepen our understandings of damage tolerance and deformation behaviors [7,8,9], but may also create new opportunities and challenges for silicon nanodevices [10,11] and silicon-based anodes [12,13]. Usually, three widely known methods are used to explore the mechanical response of silicon under strain rate effects. The first comprises theoretical efforts to conclude and predicate the rate-dependent mechanical response, such as constitutive equations [14,15,16], dislocation plasticity models [17,18] and rate response models [19]. However, the material properties of silicon, such as its widely known brittle-to-ductile transition characteristics, often make theoretical study complicated. The second is low strain rate nanomechanical experiments [20,21,22] (mostly <10^0^/s). For example, in situ tensile experiments on amorphous silicon nanowires indicated that brittle-to-ductile transitions were strongly rate-dependent [23]. Smith et al. [24] studied the compression behaviors of monocrystalline silicon, from which the rate- and orientation-dependency were discussed. The rate-dependence of hardness [22] in amorphous silicon and the effects of scribing rate on monocrystalline silicon [25] have been reported using nanoindentation tests. The promising engineering prospects given by these experimental methods have stimulated the efforts to understand the strain rate-dependency of silicon properties. However, for some unavailable experimental conditions, such as defect-free and extremely high strain rates, researchers have often tried to resort the third method: high strain rate molecular dynamics simulations (mostly >10^8^/s). Recent simulations [26,27,28,29,30,31] have shown that the amount of plasticity remains constant for brittle glassy nanowires as the strain rate decreases, but that plasticity decreases for the ductile counterparts [26]. Chen et al. [28] suggested that the tensile strength and the fracture pattern of ideal silicon nanorod were strongly rate-dependent. The rate-dependent tensile response obtained through different potentials also exhibited great consistency, namely, the tensile strength would reduce as applied strain rate decrease [28,29].

However, a huge gap in the timescale between simulations and experiments [32] always results in discrepancies in learning the rate-dependent mechanical response, especially when the deformation mechanisms show rate-dependency. To overcome this shortage of molecular dynamics methods, simulations covering timescales over ten orders of magnitude should be considered [33]. Although there are abundant literatures about the rate-dependent mechanical response of silicon, most of these studies have focused on amorphous silicon structures or surface indentation, resulting in a relatively poor understanding of some typical silicon structures, such as the monocrystalline silicon wafer and silicon nanowire [34,35]. Meanwhile, for the yield criterion of silicon, the effects of strain rate have not been systematically discussed through the aspects of equivalent stresses. The discovery of the yield criteria of typical silicon structures (single crystal, nanowire, nanotube, etc.) under strain rate effects would certainly benefit their engineering applications. Starting from these concerns, we are trying to figure out some unsolved questions regarding the rate-dependent mechanical response of silicon on the basis of present researches, especially concentrated on ignored points, such as surface effects and yield criterion.

With the aid of molecular dynamics, the rate-dependent mechanical response of two typical monocrystalline silicon structures were investigated in the present paper. Two important parameters about the rate-dependent mechanical response were calculated according to a rate response model. Meanwhile, the hidden rate-dependent deformation mechanism and yield criterion were discussed through analyzing dislocation activities and comparing equivalent stresses, respectively.

## 2. Methodologies

### 2.1. Model and Method

**Figure 1:**
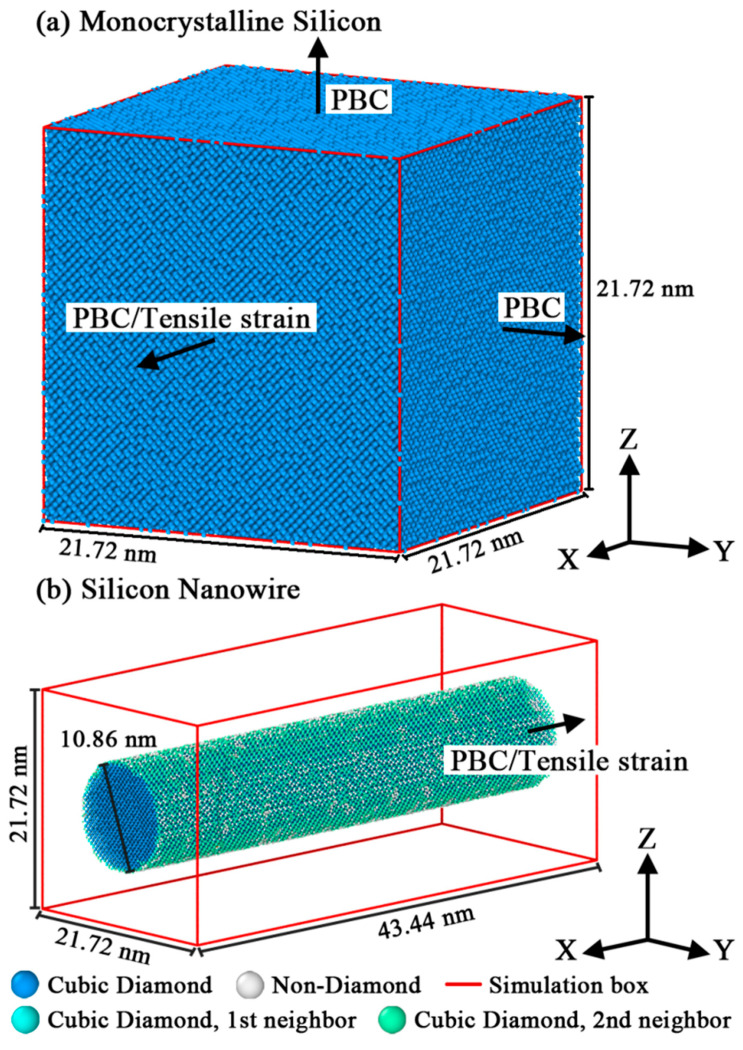
Schematic representations of the model settings.

All simulations were carried out by LAMMPS software (Large-scale Atomic/Molecular Massive Parallel Simulator, version 5 September 2018). First, a monocrystalline silicon crystal and a cylindrical silicon nanowire were generated, their schematics are shown in Figure 1. The X, Y and Z axes of the two structures corresponded to [100], [010] and [001] orientations of silicon, respectively. Periodic conditions were applied in all directions of systematic boundaries to eliminate additional surface effects.

After the establishment of these two structures, they were relaxed under a 300 K, 1 atm standard NPT (isobaric/isothermal constant number of particles, constant pressure and constant temperature) ensemble. The pressures of the X, Y and Z axes were controlled close to zero by a Berendsen [36] barostat in the relax stage. However, in the deformation process, only the pressures of the Y and Z axes were controlled close to 100 MPa by the Berendsen barostat, in order to ensure the ambient pressure. The system temperature was controlled close to 300 K via a Berendsen thermostat during the deformation process. The selection of interatomic potential is critical for the simulation results. To obtain accurate descriptions about the elastic properties of silicon, we adopted Tersoff’s approach that developed in 1988 [37]. The comparison of interatomic potentials about material properties was given in Table A1 (see Appendix A for details).

Then, for each simulation case, a uniaxial tensile strain rate $\dot{\varepsilon }$ was loaded along the X axis to commence the deformation process. Ten strain rates were simulated for each structure, which ranged from 10^−1^/ps to 5 × 10^−6^/ps (e.g., the first was 10^−1^/ps, the second was 5 × 10^−2^/ps, the third was 10^−3^/ps, etc.) to cover the possible range of molecular dynamics simulations with calculation consumption as low as possible. In addition, we used the logarithm to express the strain rate, which was intended to ensure clarity and readability.

### 2.2. Relevant Theories

In this study, a modified rate response model [19] based on FCC metals was introduced to examine the rate-dependence of silicon. For applied strain rate $\dot{\varepsilon }$, the modified model takes the form below:

(1) $$\sigma_{s}=\sigma_{quasi\text{-}static}\left[1+c\times {\left(\frac{\dot{\varepsilon }}{{\dot{\varepsilon }}_{c}}\right)}^{p}\right],$$

where *σ_s_* is the rate-dependent tensile strength and *σ_quasi-static_* is the quasi-static value (quasi-static strain rate range: 10^−17^/ps to 10^−14^/ps [38]) of tensile strength. *p* represents strain rate sensitivity (for Cu, *p* ≈ 0.64 and for Ni, *p* ≈ 0.5 [19]). ${\dot{\varepsilon }}_{c}$ is the critical strain rate. The tensile strength changes a little at low strain rate and increases rapidly after the critical strain rate. Under this condition, however, ${\dot{\varepsilon }}_{c}$ cannot be treated as the critical strain rate, because the tensile strength is no longer rate-independent. The original form of Equation (1) was first proposed by Cowper and Symonds [39], but Guo et al. [19] modified the original form by adding parameter *c*, allowing the modified model to be adjusted according to ${\dot{\varepsilon }}_{c}$. According to their article, the modified parameter *c* is defined by Equation (2):

(2) $$c=\frac{\sigma_{c}}{\sigma_{quasi\text{-}static}}-1,$$

where *σ_c_* is the critical tensile strength under the corresponding ${\dot{\varepsilon }}_{c}$. Therefore, the modified rate response model is introduced, and it will be discussed in the next section.

## 3. Results

### 3.1. Strain Rate Sensitivity

**Figure 2:**
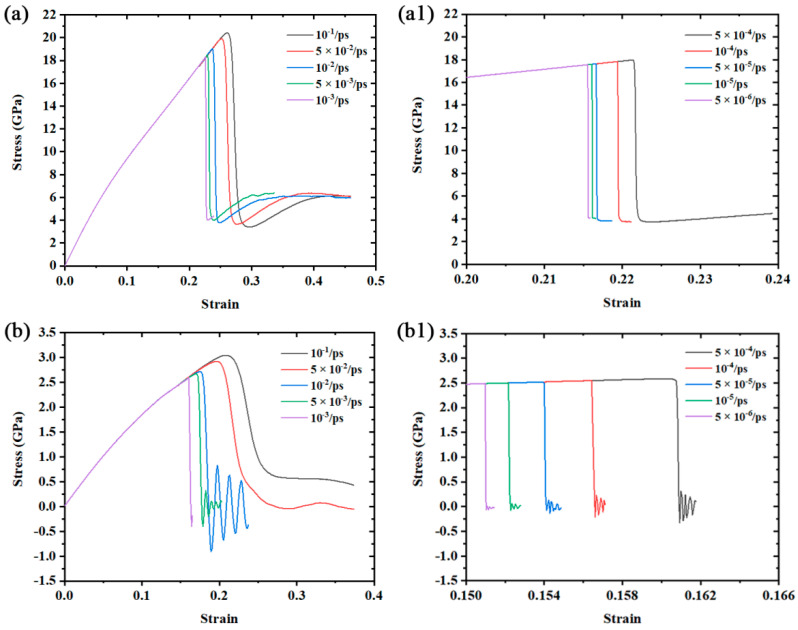
Stress–strain curves of the two structures under the simulated strain rates: (**a**) Mono-Si, strain rate range: 10^−1^/ps to 10^−3^/ps; (**a1**) Mono-Si, strain rate range: 5 × 10^−4^/ps to 5 × 10^−6^/ps; (**b**) SiNW, strain rate range: 10^−1^/ps to 10^−3^/ps; (**b1**) SiNW, strain rate range: 5 × 10^−4^/ps to 5 × 10^−6^/ps.

The tensile stress–strain curves of Mono-Si and SiNW are plotted in Figure 2a,a1,b,b1, from which we can see a smooth stress increase and then a sudden drop. Both the tensile strength and the maximum tensile strain reduce, while Young’s modulus stays roughly at 166.37 GPa for Mono-Si and 20.79 GPa for SiNW as the applied strain rate decreases from 10^−1^/ps to 5 × 10^−6^/ps. We also found that the toughness of Mono-Si is stronger than SiNW by comparing the maximum tensile strain under the same strain rate. In addition, it should be noted in Figure 2 that both Mono-Si and SiNW match the expectations of rate-dependent materials [40] under the test strain rate, according to the stress–strain curves and the variations of tensile strength. So, the theoretical rate response model introduced in Section 2.2 could be applied after obtaining ${\dot{\varepsilon }}_{c}$ and *σ_quasi-static_*.

**Figure 3:**
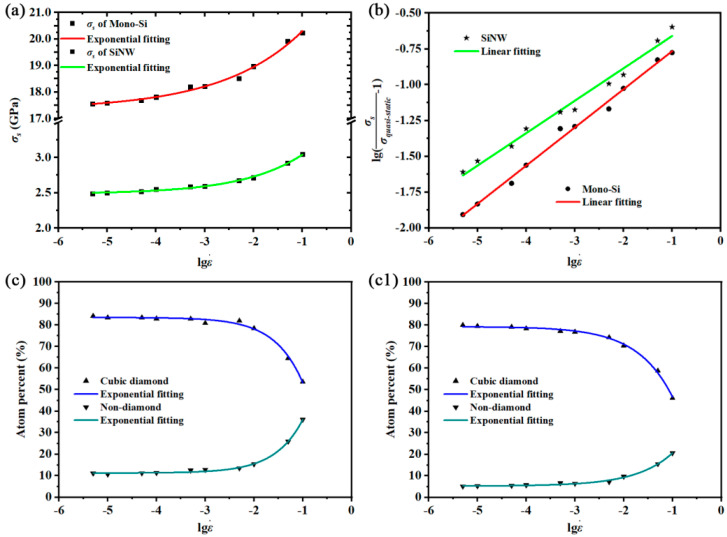
(**a**) lg$\dot{\varepsilon }$ versus *σ_s_*; (**b**) lg$\dot{\varepsilon }$ versus lg$\left(\frac{\sigma_{s}}{\sigma_{quasi\text{-}static}}-1\right)$; (**c**) variations of non-diamond atoms and cubic diamond atoms of Mono-Si after fracture; (**c1**) variations of non-diamond atoms and cubic diamond atoms of SiNW after fracture.

First, we plot lg$\dot{\varepsilon }$ versus *σ_s_* in Figure 3a to compute the quasi-static tensile strength. The exponential relation is used to fit lg$\dot{\varepsilon }$ versus *σ_s_* for both structures, and it takes the form below:

(3) $$\sigma_{s}=\sigma_{0}+A\times e^{R\times \mathrm{lg}\dot{\varepsilon }},$$

where *A* and *R* are automatically fitting parameters and *σ*_0_ represents the tensile strength under the ultra-low strain rate ($\dot{\varepsilon }\;\;\ll$ 10^−6^/ps) in this fitting. In this study, *σ*_0_ is approximately treated as *σ_quasi-static_* based on the results given in Figure 3a. Then, we can rewrite Equation (1) as:

(4) $$\mathrm{lg}\left(\frac{\sigma_{s}}{\sigma_{quasi\text{-}static}}-1\right)=p\mathrm{lg}\dot{\varepsilon }+\left(\mathrm{lg}c-p\mathrm{lg}{\dot{\varepsilon }}_{c}\right),$$

where *σ_quasi-static_* is equal to 17.335 GPa for Mono-Si and 2.43 GPa for SiNW. To figure out the strain rate sensitivity *p*, lg$\dot{\varepsilon }$ versus lg$\left(\frac{\sigma_{s}}{\sigma_{quasi\text{-}static}}-1\right)$ is plotted in Figure 3b, and the corresponding fittings show two linear relations. So, the rate sensitivity *p* is solved by calculating the slopes of linear fittings. The strain rate sensitivities of Mono-Si and SiNW are 0.267 and 0.226, respectively. However, the constant *c* and critical strain rate ${\dot{\varepsilon }}_{c}$ in the rate response model are still unknown. The calculation of ${\dot{\varepsilon }}_{c}$ is primarily considered using the structural variations. Figure 3c,c1 show the variations of cubic diamond and non-diamond atoms after fracture, which represent the undamaged structures and fractured structures, respectively. It is obvious that these structural variations follow the same trend with *σ_s_* and the values of non-diamond percent and cubic diamond percent became stable under 10^−4^/ps. Combined with the analysis about the variations of *σ_s_*, ${\dot{\varepsilon }}_{c}$ could be roughly regarded as 10^−4^/ps. In that case, constant *c* is 0.0274 (Mono-Si) and 0.0493 (SiNW) through Equation (2). After obtaining all required parameters in the rate response model, we can conclude that ${\dot{\varepsilon }}_{c}$ is the same, while *p* is different for the two silicon structures, which means the strain rate sensitivity is weakened by the additional surface effects of SiNW. In addition, the calculations of strain rate sensitivity and critical strain rate are also beneficial for predicting the rate-dependent mechanical response of these silicon structures.

### 3.2. Rate-Dependent Dislocation Activities

**Figure 4:**
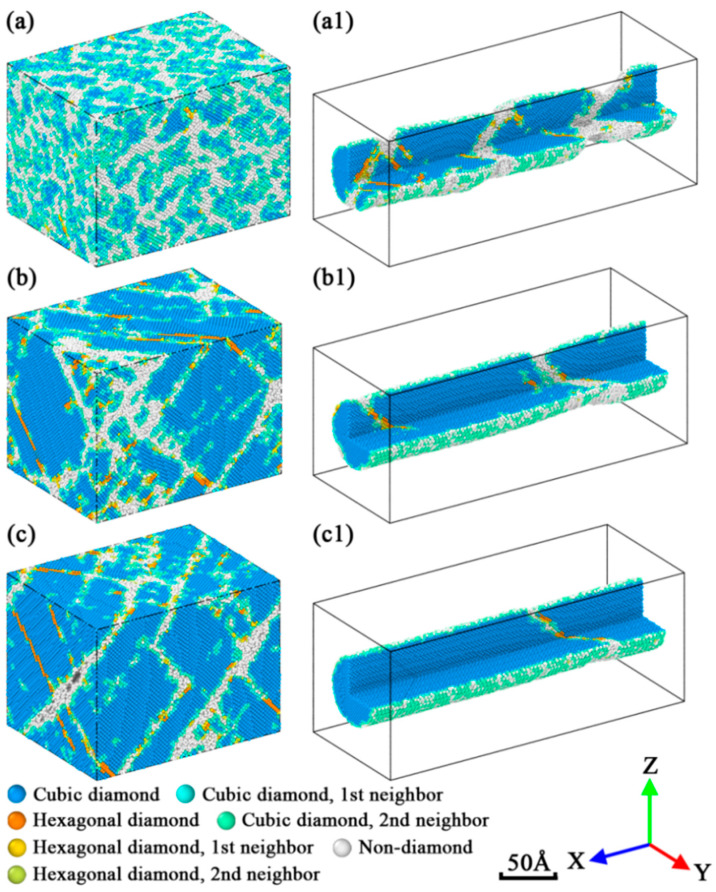
Cleavage fracture of Mono-Si under three strain rates: (**a**) 10^−2^/ps; (**b**) 10^−4^/ps; (**c**) 10^−6^/ps. Slip fracture of SiNW under three strain rates: (**a1**) 10^−2^/ps; (**b1**) 10^−4^/ps; (**c1**) 10^−6^/ps. The SiNWs were sliced to reveal their inherent structures.

The fracture patterns of two structure types are plotted in Figure 4 via Ovito [41], from which the high strain rate deformation was found activating more crystal structures in the fracture process, resulting in the increase in disordered structures after fracture. On the other hand, the fractured structures gradually decrease to a low percent once the applied strain rate is lower than the critical strain rate. If we compare the fracture patterns of Mono-Si and SiNW, it is clear that the slip fracture tends to occur in SiNW while the cleavage fracture is more likely to appear in Mono-Si. This result shows great consistency with the existing literature [42,43].

**Figure 5:**
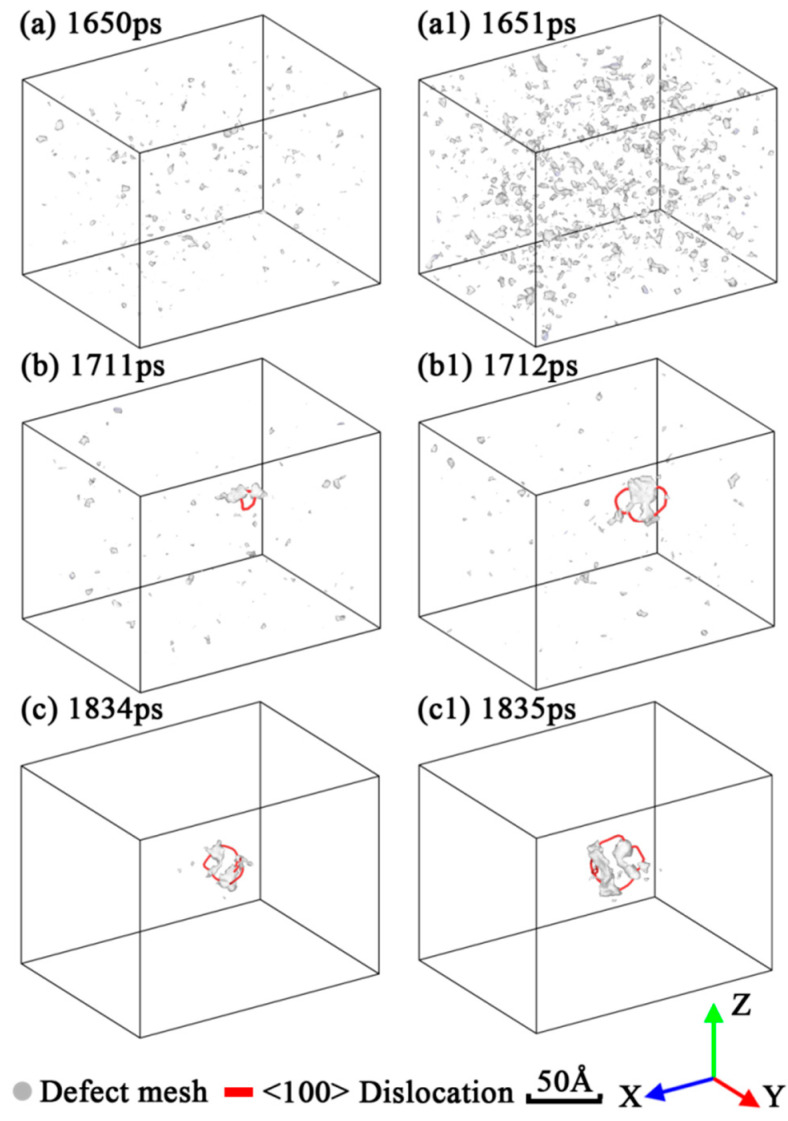
Dislocation and defect mesh in the fracture process of Mono-Si under three strain rates: (**a**,**a1**) 10^−2^/ps; (**b**,**b1**) 10^−4^/ps; (**c**,**c1**) 10^−6^/ps. The atoms were removed for clarity.

**Figure 6:**
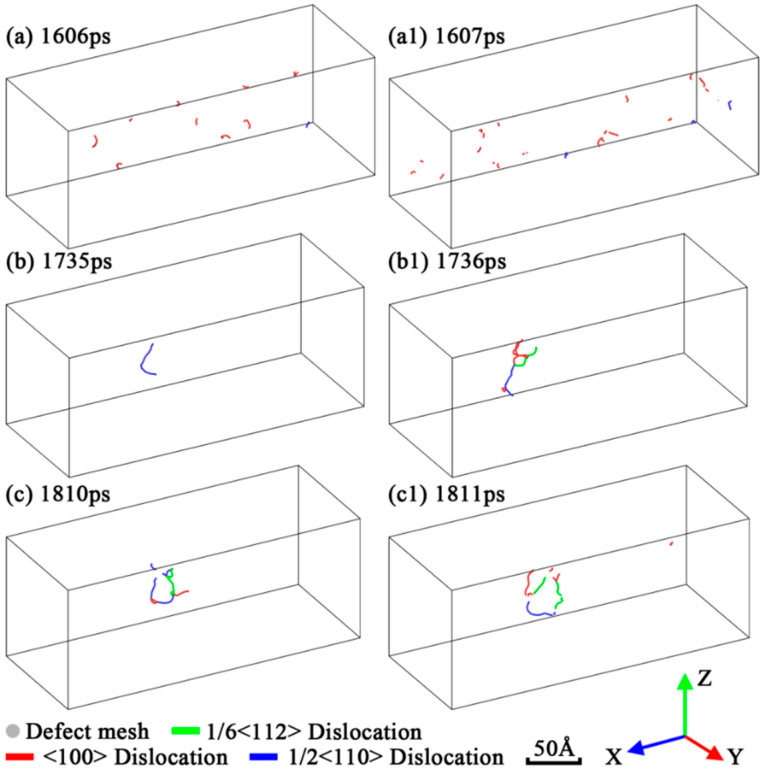
Dislocation and defect mesh in the fracture process of SiNW under three strain rates: (**a**,**a1**) 10^−2^/ps; (**b**,**b1**) 10^−4^/ps; (**c**,**c1**) 10^−6^/ps. The atoms were removed for clarity.

In order to indicate the formations of these fracture patterns and the low tensile strength under low strain rate deformation, an automated dislocation extraction (DXA) algorithm [44] was used to identify the dislocations in each simulation and plot the results in Figure 5 and Figure 6. As shown in Figure 5a,a1,c,c1, intensive small defect clusters were generated in the high strain rate deformation of Mono-Si, while only a dislocation loop and some relatively larger defect clusters were generated in the low strain rate deformation. Compared with the Mono-Si counterparts, a lot of short dislocation lines were generated on the surface of SiNW in Figure 6a,a1, but these dislocations lacked the ability for expansion or motion. On the other hand, in Figure 5b,c and Figure 6b,c, a lower strain rate allowed the dislocation-driven slip fracture to expand along the {100} cleavage planes. Therefore, for Mono-Si, it is concluded that a low strain rate may benefit dislocation activities such as nucleations and motions, resulting in the low tensile strength of the tested Mono-Si crystals. As for the SiNW, the increase in strain rate would certainly reduce its dislocation motion abilities but increase the dislocation nucleation events in its surface. Besides, low coordination and high interface energy on the SiNW surface could also benefit the dislocation nucleation in the earlier stages of stress–strain curves, indicating that the introduction of additional surfaces causes the difference of tensile strength between Mono-Si and SiNW.

### 3.3. Comparison of Equivalent Stresses

**Figure 7:**
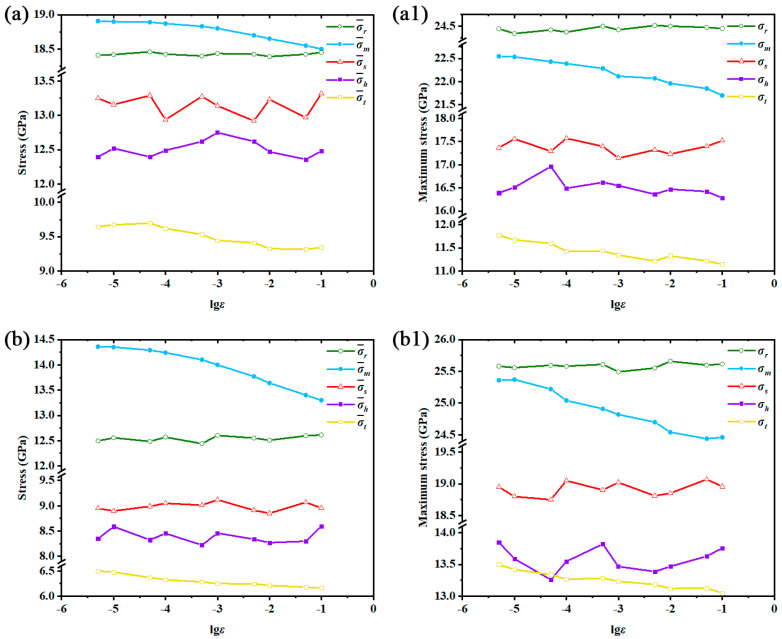
lg$\dot{\varepsilon }$ versus five equivalent stresses: (**a**) average stress level of Mono-Si; (**a1**) maximum stress level of Mono-Si; (**b**) average stress level of SiNW; (**b1**) maximum stress level of SiNW.

To explore the hidden mechanisms of the rate-dependent mechanical response, the equivalent stresses were considered according to reference [42]. However, the stress components were required in the calculation of equivalent stresses. Here, a single atom was treated as a microelement so that its stress components could be represented by Virial stress [45]. First, Virial stress tensors [46,47] were computed for each atom. The computation details of these atomic Virial stress tensors are given in Appendix B for reference. Then, five equivalent stresses were calculated using the atomic Virial stress tensors following:

(5) $$\sigma_{h}=\frac{\left(\sigma_{x}+\sigma_{y}+\sigma_{z}\right)}{3},$$

(6) $$\sigma_{r}=\sigma_{1},$$

(7) $$\sigma_{s}=\sigma_{1}-v(\sigma_{2}+\sigma_{3}),$$

(8) $$\sigma_{t}=\frac{\sigma_{1}-\sigma_{3}}{2},$$

(9) $$\sigma_{m}=\frac{1}{\sqrt{2}}\sqrt{{\left(\sigma_{x}-\sigma_{y}\right)}^{2}+{\left(\sigma_{y}-\sigma_{z}\right)}^{2}+{\left(\sigma_{z}-\sigma_{x}\right)}^{2}+6\left(\tau_{xy}^{2}+\tau_{yz}^{2}+\tau_{zx}^{2}\right)},$$

where *σ_x_*, *σ_y_*, *σ_z_*, *τ_xy_*, *τ_yz_* and *τ_xz_* are the Virial stress tensors of each atom. *σ*_1_, *σ*_2_ and *σ*_3_ are the three principal stresses computed from the atomic Virial stress tensors. *v* is the Poisson’s ratio. *σ_h_* represents the hydrostatic stress and *σ_r_* represents the maximum principal stress given by Rankine (Rankine stress). *σ_s_* represents the equivalent stress based on the maximum principal strain given by St. Venant (St. Venant stress). *σ_t_* represents the Tresca stress while *σ_m_* represents the von Mises stress. The calculations of three principal stresses and five equivalent stresses follow the methods stated in reference [48]. Thus, five equivalent stresses were computed for each atom. Since fracture is the statistical results of atomic stress conditions, the average values of these equivalent stresses are given in Figure 7, and the maximum values are also given for reference.

As shown in Figure 7, the five equivalent stresses exhibited nearly the same variations for both Mono-Si and SiNW, so they jointly shared the same internal stress conditions in the deformation process. After confirming the consistency of the two structures in the stress conditions, the corresponding yield criteria were verified by comparing the five equivalent stresses in Figure 7. It is clear that the Rankine stress, St. Venant stress and hydrostatic stress were inappropriate for describing the yield criteria of Mono-Si and SiNW under strain rate effects due to their rate-independent variations. If we compare the Tresca stress and the von Mises stress, it is obvious that the von Mises stress showed better relevance in rate-dependency than the Tresca stress, because it gradually decreased as the applied strain rate increased. The von Mises stress not only considers the combination effects of tensile stress and shear stress, but also describes the stress conditions better than Tresca stress under the critical strain rate. Thus, through the comparison of five equivalent stresses, the von Mises stress was demonstrated to be the best in describing the yield criterion under various strain rates.

**Figure 8:**
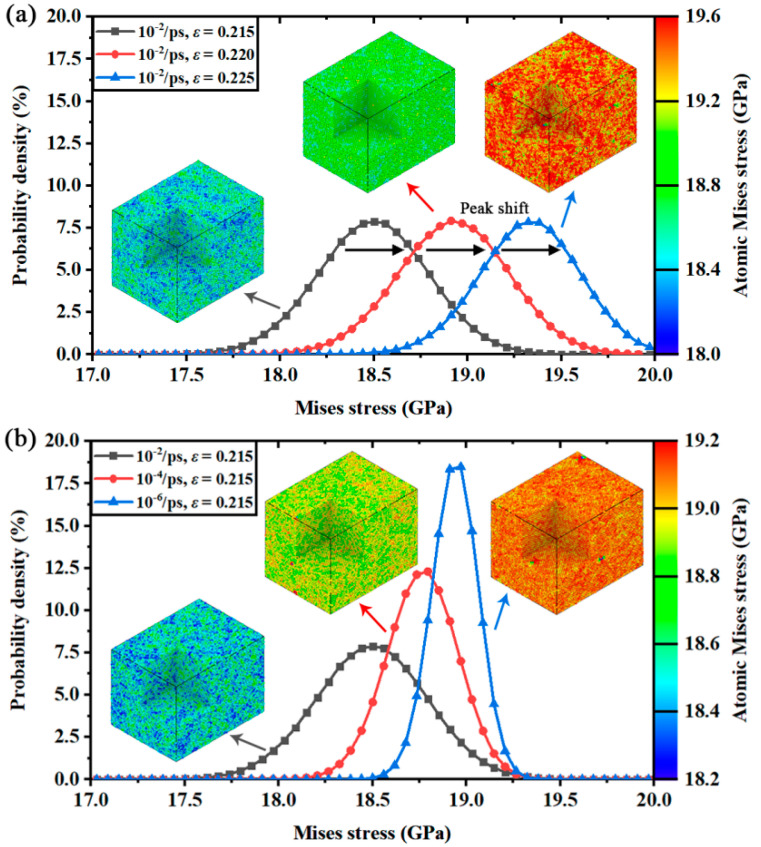
Probability density of von Mises stress distributions in Mono-Si: (**a**) at three different applied strains and equal strain rates; (**b**) at three different strain rates and equal applied strains.

**Figure 9:**
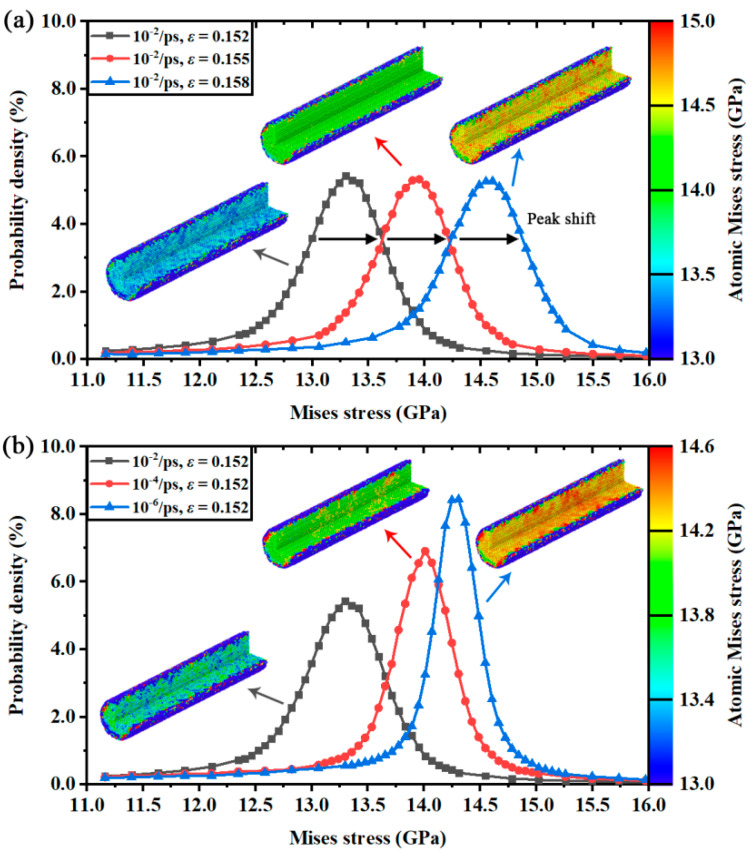
Probability density of von Mises stress distributions in SiNW: (**a**) at three different applied strains and equal strain rates; (**b**) at three different strain rates and equal applied strains.

Another conclusion that can be carried out from Figure 7a,b is that the average von Mises stress was almost linear with lg$\dot{\varepsilon }$ when the applied strain rate exceeded ${\dot{\varepsilon }}_{c}$. Then, the average von Mises stress gradually started to approach a constant value under ${\dot{\varepsilon }}_{c}$, from which ${\dot{\varepsilon }}_{c}$ acted as the division of such variations. The constant von Mises stress value could be considered as the stress threshold of fracture. Irreversible fracture is activated once the average von Mises stress reaches the threshold. In this way, the equivalent stresses and the corresponding yield criteria of the two silicon structures are analyzed and discussed under the strain rate effects. However, it is still unknown how the strain rate affects von Mises stress in the deformation process. To investigate this incomplete point, the distributions of von Mises stress are given in Figure 8 (Mono-Si) and Figure 9 (SiNW) in the form of probability density.

Obviously, the numerical distributions of von Mises stress followed the normal distribution. The stress peaks gradually shifted to a high value as the applied strain varied. Interestingly, the stress peaks exhibited different shapes under different strain rates. It seems that the atomic Virial stress was separated to a wide range of distribution interval by the high applied strain rate. Meanwhile, the width of the distribution peak (e.g., full width at half maximum) was narrowed by the low strain rate deformation. Once the stress distributions of three designated strain rates were compared at a same applied strain in Figure 8b and Figure 9b, it was found that the peak positions (average stress) were different. Accordingly, there were two parameters controlling the average von Mises stress in the deformation process. The first was the applied strain, which affected the peak positions of stress distributions. The second was the applied strain rate, which affected the peak shapes of stress distributions.

Different from the other rate-independent equivalent stresses, the average von Mises stress decreases as the applied strain rate increases. Combined with the colored subfigures about the crystal stress in Figure 8, the low strain rate deformation was found suffering a more serious von Mises stress concentration than the high strain rate deformation. Thus, the rate-dependent distributions of von Mises stress and the variations of average von Mises stress indicated an unexpected fact: compared with the high strain rate deformation, the stress fields in the low strain rate deformation had a higher possibility of exploring the potential stress threshold of fracture, or experiencing more frequent barrier crossing events [12] under the same applied strain, which could also be demonstrated by the variations of maximum von Mises stress shown in Figure 7a1,b1. Therefore, the fracture process will appear earlier in the stress–strain curves of low strain rate deformation because of the strengthened von Mises stress concentration.

## 4. Conclusions

In the present paper, two silicon structures were tested using high tensile strain rate molecular dynamics simulations. The results are concluded below:

Through a rate response model, the strain rate sensitivity and the critical strain rate of Mono-Si and SiNW were calculated, from which the extra surface of SiNW was found decrease strain rate sensitivity. However, the critical strain rate was the same for these structures, indicating that it is an inherent property which cannot be affected by surface effects. Then, the dislocation activities in the fracture process were examined. It was found that the rate-dependent dislocation nucleations and motions caused the earlier yield behaviors in the stress–strain curves of low strain rate deformation. Finally, five equivalent stresses and their descriptions on the rate-dependent mechanical response were verified; the von Mises stress was proved better than the Tresca stress in describing the yield criteria of the two silicon structures under the strain rate effects. The applicability of von Mises stress in the rate-dependent mechanical response indicates that a high strain rate not only affects internal stress fields, but also influences the elastic strain energy.

## Data Availability

Data available on request due to restrictions like data capacity. The data presented in this study are available on request from the corresponding author. The data are not publicly available due to data capacity (According to an uncompleted statistic, the related simulation data in the present paper is about at least 50 GB) which is too big to storage for the website. Please inform the corresponding author for further usage of the related simulation data.

## Appendix A

To demonstrate the reason for using Tersoff T2 potential in this study, the descriptions of various methods about the material properties were compared. These listed material properties, such as elastic constants, vacancy formation energy and bulk modulus were calculated under equilibrium state and nearly zero strain. Only the elastic properties were considered in this article due to the research topic. In Table A1, the Tersoff T2 potential was found in well accordance with the experimental results of the elastic properties. The comparisons about other properties, such as thermal properties and crystal defects, were given in the reference [49].

**Table A1:** Properties of silicon in its equilibrium structure [49] from experiment [50], quantum-mechanical (QM) methods such as density functional theory (DFT) [51], as well as tight binding (TB) [52] calculations and for various analytical potentials (Tersoff T2: Tersoff J. in 1988 [37], Tersoff T3: Tersoff J. in 1989 [53], SW: Stillinger and Weber [54], EDIP: Bazant et al. [55], DS: Dyson and Smith [56]). *a*_0_: lattice constant; *E**_C_*: cohesive energy; *B*: bulk modulus; *cij*: elastic constants; *S*: static shear modulus; *E**_V_*: vacancy formation energy; ζ: Kleinman parameter.

Properties	Expt.	QM Methods		Analytical Potentials					
		DFT	TB	Tersoff T2	Tersoff T3	SW	EDIP	ABOP	DS
*a*_0_ (Å)	5.429	5.400	5.429	5.432	5.432	5.431	5.430	5.429	5.432
*E_C_* (eV)	−4.63	−	−4.62	−4.63	−4.62	−4.63	−4.65	−4.63	−4.63
*C*_11_ (GPa)	168	159	167	166	143	162	175	167	109
*C*_12_ (GPa)	65	61	67	65	75	82	62	65	93
*C*_44_ (GPa)	80	85	75	77	69	60	71	72	38
*B* (GPa)	99	93	100	98	98	108	99	99	98
*S* (GPa)	−	111	−	119	119	117	112	111	114
*E_V_* (eV)	−	3.17	3.68	3.72	3.70	2.82	3.22	3.20	−
*ζ*	0.54	0.53	−	0.67	0.67	0.63	−	0.52	0.91

## Appendix B

The LAMMPS software supports multiple kinds of pressure tensors computation. To avoid the pressure tensors computation that cannot completely fill the periodic boundary. We adopted the atomic Virial tensors to compute the system pressure. The computation of atomic Virial tensors stated in reference [46,47] following:

(A1) $$\sigma_{i}^{Atom}=\frac{1}{V^{Atom}}\left(-mv_{i}⊗v_{i}+\frac{1}{2}\sum_{j(\neq i)}r_{ij}⊗f_{ij}\right),$$

where $\sigma_{i}^{Atom}$ represents the Virial tensors of atom *i*. *v_i_* is the velocity of this atom, *r_ij_* = *r_i_* − *r_j_* is the relative displacement between atom *i* and *j*, *f_ij_* is the interparticle force and ⊗ represents the tensor product. In the molecular dynamics simulation, the widely used Virial stress is considered equivalent to Cauchy stress. If one requires the pressure tensor *σ^System^* of a specific system, then he must average the atomic Virial tensors in the region/volume (2D/3D system) following:

(A2) $$\sigma^{System}=\frac{\sum_{i}^{N}\sigma_{i}^{atom}}{N},$$

where $\sigma_{i}^{Atom}$ is the atomic Virial tensors that previously calculated using Equation (A1). *N* is the total atom count contained in this region/volume.
